# Sociodemographic determinants and the age-education interaction in trust in physicians: A cross-sectional analysis of a nationally representative survey in China

**DOI:** 10.1097/MD.0000000000051455

**Published:** 2026-11-06

**Authors:** Chaoban Wang, Jie Tang, Jianrong Wu

**Affiliations:** a Department of Pediatric Hematology, West China Second University Hospital, Sichuan University, Chengdu, Sichuan Province, China; b Key Laboratory of Birth Defects and Related Diseases of Women and Children (Sichuan University), Ministry of Education, Chengdu, Sichuan Province, China; c Wenzhou Medical University, Wenzhou, Zhejiang Province, China.

**Keywords:** age-education interaction, China, cross-sectional study, health information sources, social determinants of health, trust in physicians

## Abstract

Trust in physicians supports effective communication and treatment adherence, but population-level determinants in China remain poorly described, and the age-trust association may vary by education. In this cross-sectional analysis of the nationally representative 2023 Chinese General Social Survey, all respondents who answered the item on trust in physicians were included (N = 5583). High versus low trust was modeled by univariable and multivariable logistic regression with backward elimination. Generalized social trust, self-reported health, and social engagement were derived by latent class analysis, and the age × education interaction was tested by likelihood ratio test. Overall, 83.9% of respondents (4683/5583) reported high trust. In the multivariable model (n = 5372), older age (adjusted odds ratio [OR] = 0.98 per year; 95% confidence interval [CI]: 0.975–0.986), middle or high school (OR = 0.63; 95% CI: 0.528–0.754), and college-or-higher education (OR = 0.75; 95% CI: 0.575–0.972) relative to primary-or-less education, and self-media use (OR = 0.72; 95% CI: 0.600–0.872) had lower odds, whereas women (OR = 1.38; 95% CI: 1.186–1.602), higher self-rated social status (OR = 1.06 per point), and high generalized social trust (OR = 2.49; 95% CI: 2.141–2.902) had higher odds. The age × education interaction was significant (*P* = .021): trust declined with age in the college-or-higher and middle-or-high-school groups but not in the primary-or-less group. Trust in physicians among Chinese adults was high overall but varied with age, education, social status, generalized social trust, and self-media use; the age-related decline was concentrated among more educated adults.

## 1. Introduction

Trust in physicians is a central element of the physician-patient relationship and an important psychological factor that shapes health-related behavior and outcomes. In many chronic conditions, trust in physicians mainly affects outcomes by improving treatment adherence. For example, among adults with type 2 diabetes, higher trust in physicians has been linked to better adherence to treatment plans, whereas its direct association with blood glucose control has been less consistent, suggesting that trust may act as a key pathway to adherence, while glycemic control is also influenced by other factors.^[1]^ In people with chronic diseases such as systemic lupus erythematosus, trust in the treating physician is also positively associated with medication adherence and expectations about health.^[2,3]^ Together, these findings highlight the importance of identifying individual characteristics related to low trust in physicians in order to detect groups at higher risk and to design interventions that strengthen the physician-patient relationship and improve disease management.

Previous studies suggest that a range of sociodemographic and psychosocial characteristics are related to the level of trust in physicians. Age and use of digital technology are among the most consistent factors. Younger patients are more likely than older patients to obtain health information from the internet and social media, whereas older patients rely more on face-to-face communication.^[4,5]^ Wider access to digital technology gives patients more ways to learn about illness and to evaluate physicians, but complex and conflicting online information may also increase uncertainty about treatment choices and weaken trust in physicians’ professional judgement. Limited social support has been linked to lower trust in physicians and poorer adherence, particularly during long-term follow-up and shared decision-making for chronic illness.^[6,7]^ As social media and other online platforms have become important sources of health information, patients are more willing to participate in decisions about their care.^[8,9]^ However, frequent reliance on online information of variable quality may lead some people to doubt formal medical advice and, in turn, reduce trust in physicians and the health care system.^[10]^

Sex and health insurance status are also important structural factors that may shape trust in physicians. Some studies have reported that men tend to give more negative ratings of physicians, whereas women place greater emphasis on interpersonal qualities such as honesty and kindness, and are more likely to feel respected and understood during consultations.^[11]^ Traditional gender roles may make men less likely to seek medical help and less willing to express vulnerability or ask for support, which can reduce opportunities to build trust with physicians.^[12,13]^ Lack of health insurance or inadequate coverage increases financial strain and perceived unfairness during care, and people without insurance or with limited coverage are more likely to mistrust health care institutions and physicians.^[14]^ In rehabilitation services and community mental health services, insurance status is closely related to service access; people who are uninsured or under-insured face more barriers to care and more negative experiences, which further undermines their trust in health care services and the health care system as a whole.^[15,16]^

China’s health care system has expanded rapidly over the past 2 decades, but crowding in tertiary hospitals means that many consultations are brief and focused on technical tasks, and patients’ experiences of communication vary widely.^[17]^ Patients therefore form judgements about physicians partly through direct contact and partly through information encountered outside the clinic. Online platforms have become a major source of health information in China, and the quality of that information varies widely.^[5,10]^ How these structural and informational changes map onto trust in physicians across social groups has not been examined at the population level.

However, most existing studies have focused on specific disease groups or particular service settings, with limited sample size and representativeness, and therefore cannot fully describe the overall level of trust in physicians and its social determinants in the general population. Evidence is especially scarce on the combined effects of digital media use, self-rated social status, and health insurance status, and on whether the association between age and trust in physicians differs across levels of education. Against this background, the present study uses nationally representative data from the 2023 wave of the Chinese General Social Survey (CGSS) to describe trust in physicians among residents in China, to examine the associations of individual characteristics and social structural factors with the level of trust in physicians, and to test the age × education interaction. These findings aim to provide empirical evidence for targeted interventions to improve the physician-patient relationship and for related policy development.

## 2. Methods

This cross-sectional study was reported according to the Strengthening the Reporting of Observational Studies in Epidemiology guidelines. Data were obtained from the 2023 wave of the CGSS, a nationally representative survey administered by Renmin University of China. The CGSS uses a multistage stratified probability sampling design to recruit adults aged 18 years or older from urban and rural areas across mainland China and collects information on demographic, socioeconomic, social, and health-related characteristics.^[18]^

The CGSS is a publicly available, de-identified dataset. Ethical approval and informed consent were obtained by the CGSS investigators at the time of data collection. Because the present study used secondary data that were publicly available and de-identified, no additional ethical approval or informed consent was required.

The 2023 CGSS dataset included 11,326 respondents. The item assessing trust in physicians (C17f) was administered only during face-to-face interviews. After excluding 5743 respondents without information on this item, 5583 participants were included in the analysis.

### 2.1. Assessment of trust in physicians

Trust in physicians was assessed using CGSS item C17f: “How much do you trust the following people: doctors?” Responses were categorized as 1 = trust completely, 2 = trust somewhat, 3 = trust not much, and 4 = do not trust at all. Responses 1 and 2 were classified as high trust, whereas responses 3 and 4 were classified as low trust. High trust was modeled as the outcome event. Among the 5583 participants, 4683 (83.9%) reported high trust and 900 (16.1%) reported low trust. The original questionnaire items and coding schemes are provided in Table 1 and Supplementary Table S1, Supplemental Digital Content 1.

**Table 1:** Definitions, measurement, and coding of the study variables.

Variable	CGSS 2023 item	Question or definition	Coding used in the analysis	Reference category
Trust in physicians (outcome)	C17f	How much do you trust the following people: doctors?	High trust = trust completely or trust somewhat (options 1–2); low trust = trust not much or do not trust at all (options 3–4). High trust was the event of interest.	Low trust
Age	A3a–A3c and A00	Date of birth and interview date	Completed years of age at interview (continuous)	–
Sex	A2	Sex	Male, female	Male
Educational attainment	A7a	What is your current highest level of education (including current education)?	Primary school or less (no schooling, literacy class or primary school); middle or high school (junior secondary, vocational high school, general high school, technical secondary school or technical school); college or higher (junior college, bachelor degree or postgraduate)	Primary school or less
Number of household members	A1	Number of other people in the household (excluding those who have separated)	Count (continuous)	–
Personal annual income	A8a	Total personal income in 2022 (yuan)	Continuous; CGSS special codes (≥9,999,996) and non-response set to missing	–
Self-rated social status	A43a	“Where do you think you stand on the social ladder at present?” (1 = lowest, 10 = highest)	Score from 1 to 10 (continuous); refused or do not know set to missing	–
Religious affiliation	A5	Religious belief	No religion, has a religion	No religion
Marital status	A69	Current marital status	With a partner, without a partner	Without a partner
Housing tenure	A128	The dwelling is rented or borrowed from a person or unit outside the family	Rented or borrowed, not rented or borrowed	Not rented or borrowed
Health insurance coverage	A61a	Current health insurance	Uninsured, public insurance, commercial insurance	Uninsured
Primary source of information	A29	Which of the above media is your main source of information?	Self-media (internet, mobile phone, computer), traditional media (newspapers, magazines, radio, television)	Traditional media
Self-reported health	A15–A17	Current physical health; health problems limiting daily activities in the past 4 wk; depressed mood in the past 4 wk	Two-class latent class analysis; good vs poor health (entropy 0.82)	Poor
Generalized social trust	A33–A36, B6, C11, C19	Seven items on whether most people can be trusted, whether others would take advantage, perceived fairness, happiness, trust in strangers and help seeking	Two-class latent class analysis; high vs low generalized trust (entropy 0.59)	Low
Social engagement	A44, A45, C1, C15c–C15f, C16a–C16c	Ten items on voting, trade-union and social-organization membership, political interest, willingness to contribute, neighborhood trust and support, lack of companionship, loneliness and perceived exclusion	Two-class latent class analysis; high vs low engagement (entropy 0.91)	Low

Potential covariates included age, sex, educational attainment, religious affiliation, household size, personal annual income, self-rated social status, housing tenure, marital status, primary source of information, health insurance coverage, self-reported health, generalized social trust, and social engagement. Detailed definitions, coding schemes, and reference categories are presented in Table 1.

Age was calculated from the date of birth and the interview date. Educational attainment was categorized as primary school or less, middle or high school, and college or higher. The middle category combined junior secondary school, vocational high school, general high school, technical secondary school, and technical school. Self-rated social status was measured on a 10-point scale. Self-reported health, generalized social trust, and social engagement were derived from multiple questionnaire items using latent class analysis.

For each of the 3 constructs, a 2-class latent class model was estimated using the expectation-maximization algorithm with full-information maximum likelihood. Fifteen random starts were used, and the solution with the highest log-likelihood was retained. Participants were assigned to the class with the highest posterior probability, and the larger class was labeled as the favorable state. Standardized entropy was 0.82 for self-reported health, 0.59 for generalized social trust, and 0.91 for social engagement. Given the heterogeneous content of the generalized social trust items, this classification was interpreted as a summary measure rather than a definitive measurement model.

### 2.2. Missing data

Missing values were observed for personal annual income (4.01%), primary source of information (2.92%), self-rated social status (0.95%), and health insurance coverage (0.93%). Missingness was negligible for the remaining variables (Supplementary Fig. S1, Supplemental Digital Content 2). Complete-case analysis was prespecified as the primary analysis. Multiple imputation by chained equations was performed as a sensitivity analysis using the mice package in R, generating 20 imputed datasets with 20 iterations per dataset. Predictive mean matching was used for continuous variables, logistic regression for binary variables, and polytomous logistic regression for categorical variables with more than 2 levels. The imputation model included all analysis variables and the outcome. Estimates were pooled using Rubin’s rules.

### 2.3. Data processing and statistical analysis

All analyses were performed using R version 4.5.2 (R Foundation for Statistical Computing, Vienna, Austria). Continuous variables are presented as mean with standard deviation or median with interquartile range (IQR), as appropriate, and categorical variables as numbers and percentages [n (%)]. Participants were stratified by high versus low trust in physicians. Between-group differences were assessed using the chi-square test, independent-samples *t* test, or Mann–Whitney *U* test, as appropriate.

Binary logistic regression was used to examine factors associated with high trust in physicians. Crude odds ratios (ORs) and 95% confidence intervals (CIs) were obtained from univariable models. Variables with *P* < .10 in univariable analyses were considered for multivariable analysis. Backward elimination based on likelihood ratio tests was then performed, while age and sex were retained a priori regardless of statistical significance. For categorical variables, indicator variables were used with reference categories specified in the tables.

Potential effect modification was assessed by adding pairwise interaction terms to the multivariable model and comparing models with and without the interaction term using likelihood ratio tests. The interaction between age and education was specified a priori as the primary interaction of interest. Other pairwise interactions among variables retained in the final model were considered exploratory and summarized as a heatmap (Fig. 1). No adjustment for multiple testing was applied to these 15 exploratory tests; the age × education interaction was prespecified, and the remaining interactions should, therefore, be regarded as hypothesis-generating.

**Figure 1.**
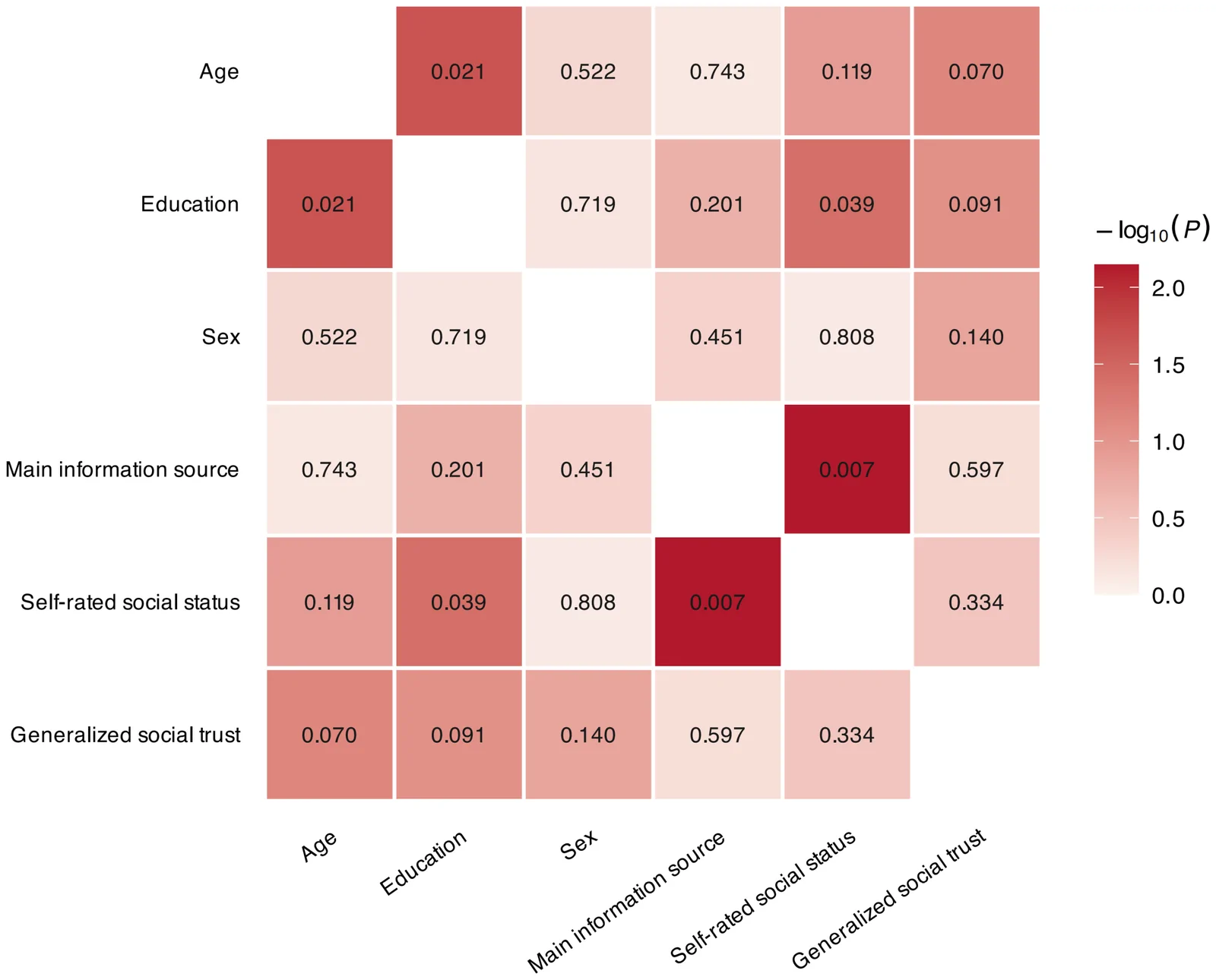
Likelihood ratio tests for all pairwise interactions among the 6 variables retained in the final multivariable logistic regression model. The outcome was high trust in physicians (vs low trust), and the model contained the main effects of age, education, sex, primary source of information, self-rated social status, and generalized social trust. For each pair of variables, a product term was added to that model, and the likelihood ratio test was used to compare the model with and without the interaction (1 degree of freedom for pairs of binary or continuous variables and 2 degrees of freedom for pairs involving educational attainment, which has 3 categories). Cell numbers are the *P* values of these tests, and the color scale encodes −log10(*P*), so that darker cells indicate smaller *P* values. Because the matrix is symmetric, each pair appears twice. The age × education interaction was specified a priori as the primary hypothesis; all pairs are shown for transparency.

For the final model containing the age × education interaction, predicted probabilities of high trust and corresponding 95% CIs were estimated across the observed age range (18–85 years) for each educational category, with other covariates fixed at their reference categories or median values. The predicted probabilities were plotted against age (Fig. 2). ORs for a 1-year increase in age were also estimated within each educational category.

**Figure 2.**
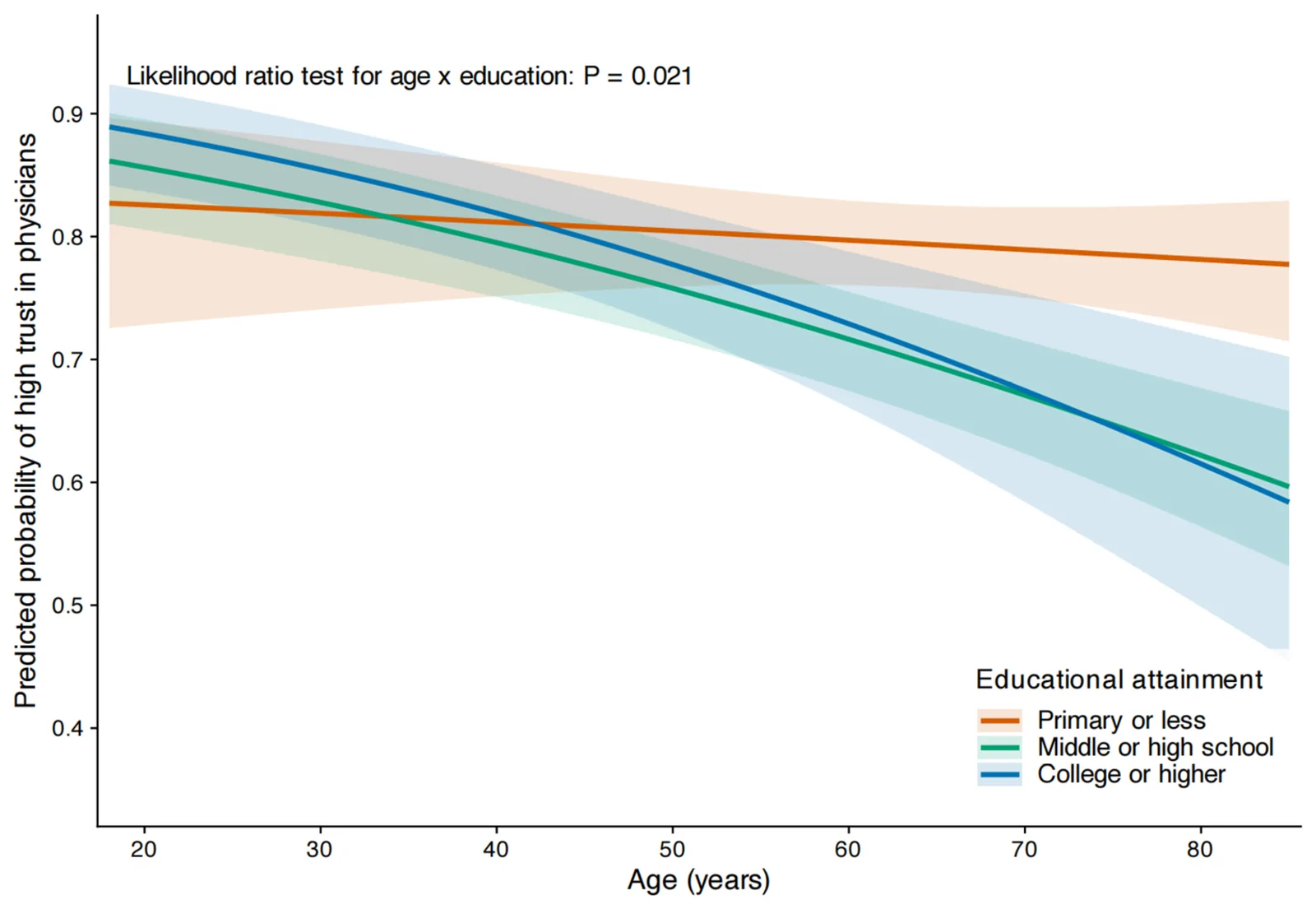
Predicted probability of high trust in physicians across the age range, by educational attainment. Probabilities were derived from the multivariable logistic regression model containing the age × education interaction and adjusted for sex, primary source of information, self-rated social status, and generalized social trust, with all covariates other than age and education fixed at their reference category or median value. The 3 curves correspond to educational levels as shown in the legend: orange, primary school or less; green, middle or high school; blue, college or higher. Shaded bands represent 95% confidence intervals. The likelihood ratio test for the age × education interaction was *P* = .021.

Two sensitivity analyses were performed: one using the full model without backward elimination and one refitting the final model on the multiply imputed datasets. All statistical tests were 2-sided, and *P* < .05 was considered statistically significant. *P* values were reported to 3 decimal places, with values < .001 reported as *P* < .001.

## 3. Results

### 3.1. Baseline characteristics of the study population

Of the 5583 participants included in the study, 900 (16.1%) reported low trust in physicians, and 4683 (83.9%) reported high trust (Table 2). Significant differences were observed between the 2 groups across several sociodemographic and psychosocial characteristics.

**Table 2:** Baseline characteristics of the study population by level of trust in physicians (N = 5583).

Characteristic	Low trust in physicians (n = 900)	High trust in physicians (n = 4683)	*P* value
Age, yr	56.28 (15.05)	53.88 (16.57)	<.001
Number of household members	2.09 (1.91)	2.29 (1.88)	.006
Self-rated social status	4.04 (1.96)	4.33 (1.86)	<.001
Personal annual income, yuan, median (IQR)	25,000 (5592–50,000)	20,000 (2500–50,000)	<.001
Sex, male	461 (51.2)	2042 (43.6)	<.001
Sex, female	439 (48.8)	2641 (56.4)	–
Religious affiliation, no religion	838 (93.1)	4295 (91.7)	.179
Religious affiliation, has a religion	62 (6.9)	388 (8.3)	–
Educational attainment, primary or less	263 (29.2)	1678 (35.8)	<.001
Educational attainment, middle or high school	506 (56.2)	2149 (45.9)	–
Educational attainment, college or higher	131 (14.6)	856 (18.3)	–
Marital status, without a partner	233 (25.9)	1230 (26.3)	.846
Marital status, with a partner	667 (74.1)	3453 (73.7)	–
Housing tenure, not rented	812 (90.2)	4296 (91.7)	.154
Housing tenure, rented	88 (9.8)	387 (8.3)	–
Health insurance coverage, uninsured	38 (4.3)	136 (2.9)	.091
Health insurance coverage, public insurance	851 (95.4)	4479 (96.6)	–
Health insurance coverage, commercial insurance	3 (0.3)	24 (0.5)	–
Primary source of information, traditional media	271 (31.1)	1563 (34.4)	.066
Primary source of information, self-media	601 (68.9)	2985 (65.6)	–
Self-reported health, poor	284 (31.6)	1387 (29.6)	.261
Self-reported health, good	616 (68.4)	3296 (70.4)	–
Generalized social trust, low	493 (54.8)	1533 (32.7)	<.001
Generalized social trust, high	407 (45.2)	3150 (67.3)	–
Social engagement, low	195 (21.7)	895 (19.1)	.085
Social engagement, high	705 (78.3)	3788 (80.9)	–

Participants with low trust were older than those reporting high trust (mean age, 56.28 vs 53.88 years; *P* < .001). The number of household members showed a small but statistically significant difference (2.09 vs 2.29; *P* = .006), whereas median personal annual income was higher in the low-trust group (25,000 yuan [IQR, 5592–50,000] vs 20,000 yuan [IQR, 2500–50,000]; *P* < .001). Individuals with low trust also reported lower self-rated social status (4.04 vs 4.33; *P* < .001). Sex distribution varied significantly: women constituted a smaller proportion of the low-trust group than of the high-trust group (48.8% vs 56.4%; *P* < .001). Religious affiliation did not differ significantly (*P* = .179). Educational attainment showed a distinct pattern (*P* < .001): the proportion of participants with primary-or-less education was lower in the low-trust group (29.2% vs 35.8%), whereas the proportion with middle or high school education was higher (56.2% vs 45.9%), and the proportion with tertiary education was somewhat lower (14.6% vs 18.3%).

Self-reported health status showed no significant group difference (*P* = .261). Housing tenure (rented or borrowed vs. not) was also comparable between groups (*P* = .154), as were marital status (*P* = .846) and health insurance coverage (*P* = .091). There was a borderline difference in the primary source of information (*P* = .066), with more individuals in the low-trust group relying on self-media. A pronounced difference was observed in generalized social trust: the low-trust group had a substantially higher proportion of individuals with low generalized trust (54.8% vs 32.7%; *P* < .001). Levels of social engagement were similar between groups (*P* = .085).

Overall, individuals with low trust in physicians tended to be older, more often male, and to have lower perceived social status and lower generalized social trust. Differences in education suggested that low trust was not concentrated among those with the least schooling, but rather reflected more complex patterns related to intermediate educational levels and broader social attitudes.

### 3.2. Factors associated with high trust in physicians

Table 3 summarizes the associations between individual characteristics and the likelihood of reporting high trust in physicians. In the univariate analysis, age was negatively associated with high trust (OR = 0.99; 95% CI: 0.986–0.995; *P* < .001), whereas female sex was positively associated with high trust (OR = 1.36; 95% CI: 1.177–1.567; *P* < .001). Compared with respondents with primary education or less, those with a middle or high school education had significantly lower odds of high trust (OR = 0.67; 95% CI: 0.566–0.783; *P* < .001), while tertiary education showed no significant association (OR = 1.02; 95% CI: 0.818–1.283; *P* = .835). Higher self-rated social status (OR = 1.08 per point; 95% CI: 1.043–1.126; *P* < .001) and high generalized social trust (OR = 2.49; 95% CI: 2.154–2.877; *P* < .001) were both associated with increased odds of high trust. A greater number of household members (OR = 1.06; 95% CI: 1.017–1.101; *P* = .005) and public insurance coverage (OR = 1.47; 95% CI: 1.019–2.123; *P* = .039) also showed positive associations, and higher social engagement was weakly associated with high trust (OR = 1.17; 95% CI: 0.983–1.394; *P* = .077), whereas reliance on self-media was marginally associated with lower trust (OR = 0.86; 95% CI: 0.737–1.006; *P* = .060). Other variables, including self-reported health, religion, marital status, housing tenure, and income, were not significant in the univariate models.

**Table 3:** Univariate and multivariable logistic regression of factors associated with high trust in physicians.

Characteristic	Crude OR (95% CI)	*P* value	Adjusted OR (95% CI)	*P* value
Age (per 1-yr increase)	0.99 (0.986–0.995)	<.001	0.98 (0.975–0.986)	<.001
Sex: female (reference: male)	1.36 (1.177–1.567)	<.001	1.38 (1.186–1.602)	<.001
Education: middle or high school (reference: primary or less)	0.67 (0.566–0.783)	<.001	0.63 (0.528–0.754)	<.001
Education: college or higher (reference: primary or less)	1.02 (0.818–1.283)	.835	0.75 (0.575–0.972)	.030
Number of household members (per additional member)	1.06 (1.017–1.101)	.005	–	–
Marital status: with a partner (reference: without)	0.98 (0.833–1.154)	.814	–	–
Religious affiliation: yes (reference: no religion)	1.22 (0.925–1.612)	.159	–	–
Housing tenure: rented (reference: not rented)	0.83 (0.652–1.060)	.136	–	–
Health insurance: public (reference: uninsured)	1.47 (1.019–2.123)	.039	–	–
Health insurance: commercial (reference: uninsured)	2.24 (0.639–7.825)	.208	–	–
Information source: self-media (reference: traditional media)	0.86 (0.737–1.006)	.060	0.72 (0.600–0.872)	<.001
Self-rated social status (per 1-point increase)	1.08 (1.043–1.126)	<.001	1.06 (1.016–1.101)	.007
Personal annual income (per 10,000 yuan)	1.000 (0.997–1.003)	.977	–	–
Self-reported health: good (reference: poor)	1.10 (0.939–1.278)	.245	–	–
Generalized social trust: high (reference: low)	2.49 (2.154–2.877)	<.001	2.49 (2.141–2.902)	<.001
Social engagement: high (reference: low)	1.17 (0.983–1.394)	.077	–	–

CI = confidence intervals, OR = odds ratio.

In the multivariable model reduced by backward elimination (n = 5372), 4 variables were retained in addition to age and sex. Older age (adjusted OR = 0.98 per year; 95% CI: 0.975–0.986; *P* < .001) and education remained independently associated with lower odds of high trust, with middle or high school education (adjusted OR = 0.63; 95% CI: 0.528–0.754; *P* < .001) and college-or-higher education (adjusted OR = 0.75; 95% CI: 0.575–0.972; *P* = .030) both showing lower odds than primary-or-less education. Female sex (adjusted OR = 1.38; 95% CI: 1.186–1.602; *P* < .001), higher self-rated social status (adjusted OR = 1.06 per point; 95% CI: 1.016–1.101; *P* = .007), and high generalized social trust (adjusted OR = 2.49; 95% CI: 2.141–2.902; *P* < .001) remained strong predictors of high trust, whereas reliance on self-media was significantly associated with lower trust after adjustment (adjusted OR = 0.72; 95% CI: 0.600–0.872; *P* < .001). The number of household members, health insurance coverage, and social engagement were removed during backward elimination. The sensitivity analyses described below produced similar estimates (Supplementary Tables S2 and S3, Supplemental Digital Content 3).

Although several associations were statistically significant, the magnitude of some estimates was modest. For example, the difference in odds of high trust between women and men corresponded to an OR of 1.38, and the association with self-rated social status corresponded to an OR of 1.06 per additional point on a 10-point scale. These estimates should, therefore, be interpreted in the context of a large nationally representative sample, in which small differences can reach statistical significance.

In the sensitivity analyses, the full model without backward elimination produced estimates close to those of the final model (Supplementary Table S2, Supplemental Digital Content 3). Household size (adjusted OR = 1.04; 95% CI: 0.996–1.082) and public insurance coverage (adjusted OR = 1.52; 95% CI: 1.033–2.223) were retained in that model but were only weakly associated with high trust, and social engagement was not associated with trust (adjusted OR = 1.09; 95% CI: 0.903–1.316). Estimates obtained after multiple imputation were almost identical to the complete-case estimates for every retained variable; for example, the adjusted OR for high generalized social trust was 2.49 in the complete-case analysis and 2.50 after imputation (Supplementary Table S3, Supplemental Digital Content 4).

### 3.3. Interaction between age and educational level

All 15 pairwise interactions among the 6 variables retained in the final multivariable model were screened with likelihood ratio tests (Fig. 1 and Supplementary Table S4, Supplemental Digital Content 5). Three pairs reached statistical significance: the interaction between the primary source of information and self-rated social status (*P* = .007), the interaction between age and education (*P* = .021), and the interaction between education and self-rated social status (*P* = .039). The age × education interaction was specified a priori as the primary hypothesis of this study and was, therefore, examined in detail in a model that also contained the main effects of all retained covariates.

In the interaction model, using college-or-higher education as the reference category, the associations observed in the main-effects model were largely retained (Table 4). Age was inversely associated with high trust among respondents with college-or-higher education (adjusted OR = 0.97 per year; 95% CI: 0.964–0.985; *P* < .001) and among those with middle or high school education (adjusted OR = 0.98 per year; 95% CI: 0.972–0.986; *P* < .001), whereas no association with age was observed among respondents with primary-or-less education (adjusted OR = 1.00 per year; 95% CI: 0.983–1.007; *P* = .448). The ratio of the age ORs relative to college-or-higher education was 1.02 (95% CI: 1.005–1.038; *P* = .010) for primary-or-less education and 1.00 (95% CI: 0.992–1.018; *P* = .490) for middle or high school education, indicating that the age-related decline in trust was significantly less pronounced among respondents with primary-or-less education than among those with college-or-higher education.

**Table 4:** Multivariable logistic regression model with the age × education interaction N=5,372.

Parameter	Adjusted OR (95% CI)	*P* value
Age (per 1-year increase), college or higher education	0.974 (0.964–0.985)	<.001
Age (per 1-year increase), middle or high school education	0.979 (0.972–0.986)	<.001
Age (per 1-year increase), primary or less education	0.995 (0.983–1.007)	.448
Interaction between age and education (likelihood ratio test)	–	.021
Ratio of age ORs, middle or high school vs college or higher	1.005 (0.992–1.018)	.490
Ratio of age ORs, primary or less vs college or higher	1.022 (1.005–1.038)	.010
Sex: female (reference: male)	1.373 (1.181–1.597)	<.001
Information source: self-media (reference: traditional media)	0.749 (0.621–0.904)	.003
Self-rated social status (per 1-point increase)	1.057 (1.015–1.101)	.007
Generalized social trust: high (reference: low)	2.475 (2.126–2.882)	<.001

Estimates for the covariates are from the model that also contains the age × education interaction term.

CI = confidence intervals, OR = odds ratio.

Predicted probability curves derived from the interaction model (Fig. 2) show the same pattern. Among respondents with college-or-higher education, the predicted probability of high trust fell from 0.87 (95% CI: 0.82–0.91) at age 25 to 0.65 (95% CI: 0.54–0.74) at age 75, and among those with middle or high school education, it fell from 0.84 (95% CI: 0.79–0.88) to 0.65 (95% CI: 0.59–0.70) over the same age range, whereas among respondents with primary-or-less education, it remained essentially unchanged (0.82 [95% CI: 0.73–0.89] at age 25 and 0.79 [95% CI: 0.74–0.82] at age 75). The 3 curves in Figure 2 correspond to the 3 educational categories shown in the legend (orange, primary school or less; green, middle or high school; blue, college or higher), and the shaded bands represent 95% CIs.

The same model also indicated that the association between the primary source of information and trust differed by self-rated social status (likelihood ratio *P* = .007): the lower odds of high trust associated with reliance on self-media were confined to respondents with lower self-rated social status. This exploratory finding is reported for completeness but was not specified a priori and requires confirmation in independent samples (Fig. 1 and Supplementary Table S4, Supplemental Digital Content 5).

## 4. Discussion

This study, based on nationally representative data from the 2023 wave of the CGSS, examined the associations between sociodemographic characteristics, social structural factors, and trust in physicians among Chinese residents, and tested whether the association between age and trust depends on educational attainment. The results indicate that overall trust in physicians is relatively high in China, but that it varies substantially across population subgroups. Increasing age was associated with lower trust among more educated respondents, whereas women, individuals with higher perceived social status, and those with higher generalized social trust were more likely to report high trust in physicians. In contrast, reliance on self-media as the primary source of information was associated with lower trust. The interaction between age and education was statistically significant but moderate in size, indicating that the mechanisms underlying trust formation are heterogeneous across social groups.

These findings are partly consistent with international research. Previous studies have suggested that older adults’ expectations towards the healthcare system, cumulative medical experiences, and reduced adaptability to new technologies may influence their trust in physicians. Nonetheless, some research has reported higher trust among older populations due to greater reliance on medical professionals, whereas our study found an opposite pattern among more educated respondents, implying that institutional changes, disparities in health resources, and varying medical burdens across generations in China may produce different trajectories of trust accumulation.^[19,20]^ Additionally, women demonstrated higher trust in physicians, aligning with literature showing that women tend to place greater value on communication quality and are more willing to articulate health-related needs.^[21,22]^ Although this difference was statistically robust in our large sample, its magnitude was moderate, corresponding to an adjusted OR of 1.38, and it should not be interpreted as indicating a fundamental difference between men and women in the propensity to trust physicians.

Evidence from China remains limited and comes mainly from single institutions or defined patient groups. Studies of patients with hypertension attending rural primary care facilities and of users of family doctor contract services reported that continuity of care and communication quality were the strongest correlates of trust,^[13,20]^ and a national analysis found that longer internet use was associated with lower trust in doctors.^[5]^ The present results are consistent with these findings and extend them in 2 respects: generalized social trust was the strongest correlate in a nationally representative sample, and the association between age and trust depended on educational attainment, a pattern that single-site studies are unlikely to detect.

Our findings also show that generalized social trust is one of the strongest correlates of physician trust, which is consistent with social capital theory: individuals’ overall trust in other members of society and in institutions strongly shapes their trust in professionals.^[23]^ This reinforces the notion that physician trust is not solely a product of interactions within the clinical encounter but is embedded within a broader context of societal trust. In the context of rapid social transition and increasingly fragmented information environments in China, generalized social trust may play an even more critical role in shaping how individuals view medical professionals.^[24,25]^

The negative association between self-media use and physician trust highlights the influence of the digital information environment. Compared with traditional media, self-media content tends to be more fragmented, emotionally charged, and lacking professional review, which may lead to uneven quality of health information, overamplification or distortion of medical risks, and, consequently, weakened authority of professional medical advice. Recent studies similarly indicate that exposure to online health information is linked to medical skepticism, poor adherence, and excessive self-diagnosis.^[5]^ Our exploratory analysis also suggested that this association was concentrated among respondents with lower self-rated social status, although this finding requires confirmation in independent samples. Thus, the self-media ecosystem may have become a pivotal structural factor in shaping public trust in medicine.

The most theoretically relevant finding of this study is the interaction between age and education. Trust declined with age among respondents with college-or-higher and middle or high school education but was stable across the age range among respondents with primary-or-less education. Several mechanisms may explain this pattern. People with more education generally possess stronger critical information-processing skills and may therefore be more inclined to question medical advice^[26]^; they also have greater exposure to digital information and are more likely to encounter controversial health content online; and expectations for medical care tend to be higher among more educated people, so that the gap between expectations and actual care may widen with age. Prior research has shown that higher education enhances health information-seeking ability and autonomous decision-making, yet these traits may paradoxically undermine trust when individuals face complex or uncertain medical information. It should be noted that the interaction was moderate in magnitude (likelihood ratio *P* = .021), so the present findings extend existing literature on the education-trust relationship rather than establishing a strong stratification effect.

The stability of trust across the age range among respondents with primary-or-less education also requires explanation. Patients in this group may have fewer alternative sources of health information, may rely on clinicians with whom they have long-standing relationships, and may judge doctors mainly through personal experience rather than through public debate about medical care; each of these mechanisms would tend to decouple trust from age. The gradual rather than abrupt decline among more educated respondents argues against a purely generational explanation and is more consistent with cumulative exposure to critical information about health care across the life course.

Health insurance coverage was associated with higher trust in the model that retained all covariates, but it was removed during backward elimination, and its association in the full model was weak. This is consistent with the observation that public insurance now covers the great majority of the Chinese population and therefore no longer differentiates strongly between patients, although the small number of uninsured and commercially insured respondents limits the precision of this estimate.

The findings have several policy implications. Efforts to improve physician trust should go beyond enhancing doctor-patient communication and incorporate broader interventions targeting societal trust foundations, digital health information governance, and public health literacy. In particular, it is essential to strengthen systems for disseminating credible health information and to ensure stricter regulation of self-media health content.^[27,28]^ Tailored strategies are also necessary, for example, improving older adults’ health literacy and digital adaptability, guiding more educated groups towards evidence-based risk cognition, and enhancing transparency within the health care system to foster greater overall trust.

Because trust was highest among respondents with the least schooling, strategies that target only low-literacy groups are unlikely to reach the groups in which trust appears to erode. Communication that acknowledges uncertainty, explains the limits of medical evidence, and is delivered by credible professional bodies may be more effective for more educated patients than general encouragement to trust doctors.

This study has limitations. First, as a cross-sectional analysis, causal relationships cannot be inferred; future studies using longitudinal or experimental designs are warranted. Second, trust in physicians was self-reported, which may introduce subjective bias. Third, the CGSS data lack detailed information on medical encounters or healthcare quality, making it difficult to disentangle the relative contributions of structural factors versus direct service experiences. Fourth, several constructs in the present study were measured with a limited number of items. Self-rated social status and the primary source of information were each captured by a single questionnaire item, and although self-reported health, generalized social trust, and social engagement were derived from multiple items by latent class analysis, the class solution for generalized social trust combined heterogeneous items and showed only modest class separation (entropy 0.59). These measurement limitations reduce the precision of the corresponding estimates and may attenuate or distort the associations observed. Future studies using validated multi-item instruments for social status, trust, and information exposure are needed. Fifth, the measurement of self-media exposure was relatively crude, distinguishing only the primary source of information rather than the amount, content, or quality of exposure; future research incorporating digital behavior data could provide more precise assessments. Finally, the interaction between age and education was identified in a single cross-sectional wave and was moderate in magnitude; replication in independent samples and in longitudinal designs is required before the pattern is used to guide targeted interventions.

## 5. Conclusions

Trust in physicians among Chinese adults was high overall but varied systematically with age, sex, education, self-rated social status, generalized social trust, and reliance on self-media for health information. The age-related decline in trust was concentrated among more educated adults, whereas trust was stable across the age range among respondents with primary-or-less education. Because physician trust is shaped not only within the medical system but also by broader informational and social trust structures, interventions should combine better regulation of online health information with communication strategies tailored to the groups in which trust appears to be eroding.^[17,29]^

## Acknowledgments

The authors thank the National Survey Research Center at Renmin University of China for making the CGSS data publicly available. No individual is named in this section, so no additional permission for naming is required.

## Author contributions

**Conceptualization:** Chaoban Wang.

**Data curation:** Chaoban Wang, Jie Tang.

**Funding acquisition:** Jianrong Wu.

**Supervision:** Jianrong Wu.

**Writing – original draft:** Jie Tang, Jianrong Wu.










