# Supplementary material for: Sociodemographic determinants and the age-education interaction in trust in physicians: A cross-sectional analysis of a nationally representative survey in China
Source: Medicine (Baltimore). 2026 Nov 6;105(45):e51455. doi: 10.1097/MD.0000000000051455 (PMC13747616; doi:10.1097/MD.0000000000051455)
Supplement: Supplementary file 1 [file medi-105-e51455-s001.docx]

Table S1. Questionnaire items used to construct the study variables

| **Variable** | **CGSS item** | **Question wording** | **Original response options** |
| --- | --- | --- | --- |
| Trust in physicians (outcome) | C17f | How much do you trust the following people? Doctors | 1 = trust completely; 2 = trust somewhat; 3 = trust not much; 4 = do not trust at all. Administered in the face-to-face survey only. |
| Sex | A2 | Sex | 1 = male; 2 = female |
| Date of birth | A3a-A3c | Date of birth | Year, month, day |
| Interview date | A00 | Interview start time | Date and time |
| Educational attainment | A7a | What is your current highest level of education (including current education)? | Original item: 1 = no schooling; 2 = private school or literacy class; 3 = primary school; 4 = junior secondary school; 5 = vocational high school; 6 = general high school; 7 = technical secondary school; 8 = technical school; 9-10 = junior college; 11-12 = bachelor degree; 13 = postgraduate; 14 = other. Collapsed for analysis: 1-3 = primary school or less, 4-8 = middle or high school, 9-14 = college or higher |
| Number of household members | A1 | Besides you, how many other people are there in your family (excluding those who have separated from you)? | Number of people |
| Personal annual income | A8a | What is your personal total income for the whole of last year (2022)? | Yuan; 9,999,996 = income above one million; 9,999,997 = not applicable; 9,999,998 = do not know; 9,999,999 = refused |
| Self-rated social status | A43a | Where do you think you stand on the social ladder at present? | 1-10 points; -3 = refused; -2 = do not know |
| Religious affiliation | A5 | Religious belief | 1 = no religion; 2 = other |
| Marital status | A69 | Your current marital status | 0 = single; 1 = have a partner (recoded from the original categories) |
| Housing tenure | A128 | The property right of your current house belongs to an individual or unit other than your family or relatives; the house is rented (borrowed) | 1 = yes; 2 = no; -3 = refused; -2 = do not know |
| Health insurance coverage | A61a | Are you currently covered by the following insurance? | 0 = did not participate; 1 = public insurance; 2 = commercial insurance |
| Primary source of information | A29 | Which of the above media is your main source of information? | Public media: newspapers, magazines, radio, television; self-media: internet (mobile phone, computer, tablet, smart wearable device) |
| Self-reported health, item 1 | A15 | How do you feel about your current physical health? | 0 = very unhealthy or relatively unhealthy; 1 = so-so; 2 = fairly healthy or very healthy |
| Self-reported health, item 2 | A16 | In the past four weeks, how often have health problems affected your work or other daily activities? | 0 = always or often; 1 = sometimes; 2 = rarely or never |
| Self-reported health, item 3 | A17 | During the past four weeks, how often have you felt depressed or downhearted? | 0 = always or often; 1 = sometimes; 2 = rarely or never |
| Generalized social trust, item 1 | A33 | Generally speaking, do you agree that most people in this society can be trusted? | 0 = disagree; 1 = neutral; 2 = agree |
| Generalized social trust, item 2 | A34 | On the whole, do you agree that in this society, if you are not careful, others will try to take advantage of you? | 0 = agree; 1 = neutral; 2 = disagree |
| Generalized social trust, item 3 | A35 | Generally speaking, do you think society is unfair? | 0 = unfair; 1 = neutral; 2 = fair |
| Generalized social trust, item 4 | A36 | Generally speaking, do you think your life is happy? | 0 = unhappy; 1 = neutral; 2 = happy |
| Generalized social trust, item 5 | B6 | Generally speaking, do you trust strangers in society now? | 0 = distrust; 1 = neutral; 2 = trust |
| Generalized social trust, item 6 | C11 | Do you ask anyone for help when you need it? | 0 = did not seek help from anyone; 1 = seek help from others |
| Generalized social trust, item 7 | C19 | In general, do you think most people can be trusted? | 0 = distrust; 1 = trust |
| Social engagement, item 1 | A44 | Did you vote in the last neighbourhood committee or village committee election? | 0 = not eligible to vote; 1 = no; 2 = yes |
| Social engagement, item 2 | A45 | Are you a member of the trade union? | 0 = never; 1 = was, but not now; 2 = yes |
| Social engagement, item 3 | C1 | Are you involved in social organizations? | 0 = did not participate; 1 = member but not active; 2 = member and active |
| Social engagement, items 4-5 | C15c, C15d | Do you agree? I am personally interested in politics; I want to contribute to society | 0 = disagree; 1 = agree |
| Social engagement, items 6-7 | C15e, C15f | Do you agree? Neighbours care for each other; when I need something, the neighbours are happy to help me | 0 = disagree; 1 = agree |
| Social engagement, items 8-10 | C16a, C16b, C16c | In the past four weeks, how often have you felt a lack of company, felt lonely, or felt that you were being excluded? | 0 = often or very often; 1 = neutral; 2 = rarely or never |
