# Supplementary material for: Sociodemographic determinants and the age-education interaction in trust in physicians: A cross-sectional analysis of a nationally representative survey in China
Source: Medicine (Baltimore). 2026 Nov 6;105(45):e51455. doi: 10.1097/MD.0000000000051455 (PMC13747616; doi:10.1097/MD.0000000000051455)
Supplement: Supplementary file 2 [file medi-105-e51455-s003.docx]

Table S2. Sensitivity analysis: full multivariable model without stepwise elimination

| **Characteristic** | **Adjusted OR (95% CI)** | **P value** |
| --- | --- | --- |
| Age (per 1-year increase) | 0.98 (0.975-0.987) | <0.001 |
| Sex: female (reference: male) | 1.38 (1.190-1.610) | <0.001 |
| Education: middle or high school (reference: primary or less) | 0.63 (0.526-0.754) | <0.001 |
| Education: college or higher (reference: primary or less) | 0.76 (0.580-0.991) | 0.042 |
| Number of household members (per additional member) | 1.04 (0.996-1.082) | 0.079 |
| Health insurance: public (reference: uninsured) | 1.52 (1.033-2.223) | 0.033 |
| Health insurance: commercial (reference: uninsured) | 2.40 (0.666-8.632) | 0.181 |
| Information source: self-media (reference: traditional media) | 0.73 (0.601-0.875) | <0.001 |
| Self-rated social status (per 1-point increase) | 1.06 (1.016-1.102) | 0.006 |
| Generalized social trust: high (reference: low) | 2.46 (2.114-2.871) | <0.001 |
| Social engagement: high (reference: low) | 1.09 (0.903-1.316) | 0.368 |
