# Supplementary material for: Sociodemographic determinants and the age-education interaction in trust in physicians: A cross-sectional analysis of a nationally representative survey in China
Source: Medicine (Baltimore). 2026 Nov 6;105(45):e51455. doi: 10.1097/MD.0000000000051455 (PMC13747616; doi:10.1097/MD.0000000000051455)
Supplement: Supplementary file 3 [file medi-105-e51455-s004.docx]

**Table S3. Sensitivity analysis: multiple imputation (m = 20) with Rubin's rules**

| **Characteristic** | **Pooled adjusted OR (95% CI)** | **P value** |
| --- | --- | --- |
| Age (per 1-year increase) | 0.98 (0.976-0.987) | <0.001 |
| Education: middle or high school (reference: primary or less) | 0.64 (0.540-0.763) | <0.001 |
| Education: college or higher (reference: primary or less) | 0.76 (0.590-0.989) | 0.041 |
| Sex: female (reference: male) | 1.37 (1.183-1.589) | <0.001 |
| Information source: self-media (reference: traditional media) | 0.73 (0.602-0.873) | <0.001 |
| Self-rated social status (per 1-point increase) | 1.05 (1.007-1.091) | 0.020 |
| Generalized social trust: high (reference: low) | 2.50 (2.154-2.900) | <0.001 |
