# Supplementary material for: Sociodemographic determinants and the age-education interaction in trust in physicians: A cross-sectional analysis of a nationally representative survey in China
Source: Medicine (Baltimore). 2026 Nov 6;105(45):e51455. doi: 10.1097/MD.0000000000051455 (PMC13747616; doi:10.1097/MD.0000000000051455)
Supplement: Supplementary file 4 [file medi-105-e51455-s005.docx]

**Table S4. Pairwise interaction tests among the variables retained in the final model**

| **Variables compared** | **Likelihood ratio chi-square** | **df** | **P value** |
| --- | --- | --- | --- |
| Primary source of information x Self-rated social status | 7.23 | 1 | 0.007 |
| Age x Educational attainment | 7.71 | 2 | 0.021 |
| Educational attainment x Self-rated social status | 6.46 | 2 | 0.039 |
| Age x Generalized social trust | 3.27 | 1 | 0.070 |
| Educational attainment x Generalized social trust | 4.78 | 2 | 0.091 |
| Age x Self-rated social status | 2.43 | 1 | 0.119 |
| Sex x Generalized social trust | 2.18 | 1 | 0.140 |
| Educational attainment x Primary source of information | 3.21 | 2 | 0.201 |
| Self-rated social status x Generalized social trust | 0.93 | 1 | 0.334 |
| Sex x Primary source of information | 0.57 | 1 | 0.451 |
| Age x Sex | 0.41 | 1 | 0.522 |
| Primary source of information x Generalized social trust | 0.28 | 1 | 0.597 |
| Educational attainment x Sex | 0.66 | 2 | 0.719 |
| Age x Primary source of information | 0.11 | 1 | 0.743 |
| Sex x Self-rated social status | 0.06 | 1 | 0.808 |

Likelihood ratio tests comparing the multivariable model with and without each pairwise product term. df = 1 for pairs of binary or continuous variables and df = 2 for pairs that include educational attainment (3 categories).
